# Identification and in-depth characterization of clinical isolates of *Peribacillus frigoritolerans*

**DOI:** 10.1128/spectrum.00245-26

**Published:** 2026-07-15

**Authors:** Marco Calvigioni, Virginia Rossi, Francesco Celandroni, Simona Barnini, Antonella Lupetti, Diletta Mazzantini, Emilia Ghelardi

**Affiliations:** 1 Department of Translational Research and New Technologies in Medicine and Surgery, University of Pisa 684774, Pisa, Italy; 2 Azienda Ospedaliero-Universitaria Pisana 9257, Pisa, Italy; Universita degli Studi dell'Insubria, Varese, Italy

**Keywords:** *Peribacillus frigoritolerans*, identification, microbiological characterization, virulence potential, antibiotic resistance, genome analysis

## Abstract

*Peribacillus frigoritolerans* is a bacterial species commonly found in the environment and used as a plant-growth promoter and biocontrol agent in agriculture. Recent evidence has proven that *Peribacillus* spp. are also able to cause severe infections in humans, thus emerging as new human pathogens. In this study, for the first time, 10 *P. frigoritolerans* strains were isolated from human samples (both superficial and sterile deep body sites) and characterized in terms of morphology, lifestyle, genetics, and virulence. The molecular identification by MALDI-TOF mass spectrometry and 16S rRNA gene sequencing was inconclusive, while whole-genome sequencing was effective in properly identifying isolates within the species *P. frigoritolerans*. The pangenome analysis provided an overview of the virulence potential of *P. frigoritolerans*, revealing the presence of genes involved in antibiotic resistance and toxin/exoenzyme production. Phenotypically, the strains displayed different features and behaviors, indicating strain-specific properties and high intra-species variability. A part of the strains exhibited virulence factors, being able to swim and swarm, form biofilms, and produce enzymes and toxins. Antibiotic susceptibility testing revealed resistance to ampicillin for all strains and resistance to erythromycin and clindamycin for some of them. Antimicrobial activity against Gram-positive bacteria and fungi was demonstrated, further corroborating the presence of putative bacteriocin/antimicrobial peptide-encoding genes. An association between the overall virulence potential and infection site/severity was hypothesized. Altogether, these findings highlight the extreme diversity within the species, reveal the strain-dependent pathogenic potential of *P. frigoritolerans*, and support its role as a candidate human pathogen.

**IMPORTANCE:**

This study provides insights into the infectious role of *Peribacillus frigoritolerans*, an almost unknown bacterial species with agrobiotechnological potential but no history of human infections. This is the first report of *P. frigoritolerans* isolation from human clinical samples. Ten *P. frigorit*-olerans strains were herein characterized for their morphology, lifestyle, genetics, and virulence, highlighting an extreme intra-species variability and the potential to act as pathogens in humans. Importantly, this study points out the need for unconventional methods for proper identification of this species, since traditional techniques result inconclusive. Resistance to commonly prescribed antibiotics was also evidenced, confirming the importance of antimicrobial testing on clinical iso-lates. This study lays the foundation for a more in-depth characterization of *Peribacillus* spp. in the clinical context.

## INTRODUCTION

The genus *Peribacillus* consists of Gram-positive, rod-shaped, spore-forming bacteria, commonly found in the environment, in the soil, and as part of the plant-associated microbiota. The conception of this novel group, separated from the genus *Bacillus*, was suggested in 2020 after a phylogenetic comparative analysis of 352 genomes of members of the *Bacillaceae* family (1). According to the List of Prokaryotic names with Standing in Nomenclature (LPSN), 25 species are currently recognized within the genus *Peribacillus* (2), including the type species *Peribacillus simplex* and *Peribacillus frigoritolerans*, the latter previously miscategorized in the genus *Brevibacterium* (3). Some members of the genus *Peribacillus*, including *P. frigoritolerans*, constitute the *Peribacillus simplex* clade (1), in which discrimination of species with canonical methods is challenging due to high similarities in protein contents and 16S rRNA gene sequences (4).

The characterization of environmental *Peribacillus* strains led to crucial insights into their biotechnological and agroecological potential. In fact, members of the genus *Peribacillus* show a very versatile metabolism and are adapted to survive in harsh environments, often producing siderophores and high-performing enzymes that may be useful for industrial and biotechnological applications (5–8). Moreover, *Peribacillus* shows marked plant-growth promoting activity, as well as protective effects on plants against stress conditions and infections (9–14), thus making strains available in the agricultural market as biocontrol agents, plant health promoters, and biostimulants (10, 15).

Little is known about the pathogenic role of *Peribacillus* species in humans and animals. The only available case reports identified *P. simplex* as the causative agent of brain abscess (16), penetrating trauma infection (17), and pyonephrosis (18). The isolation of *P. frigoritolerans* from human samples was never reported before. In the present study, 10 *P. frigoritolerans* strains isolated from human specimens in the clinical context were characterized for their morphology, lifestyle, genetics, and virulence, as well as for their ability to produce antimicrobial compounds, to confirm or reject them as potential pathogens.

## MATERIALS AND METHODS

### Microbial strains

Ten *Peribacillus* strains were isolated from human specimens submitted to the Microbiology Unit of the Azienda Ospedaliero Universitaria Pisana, Pisa, Italy, during the routine clinical workflow over a 12-month period. Strains were isolated from both superficial and deep body sites, as well as from long-term devices (Table 1), in pure culture or as prevalent microorganisms compared to other commensals, depending on the nature of the sample and according to hospital procedures. With the exception of patients from whom strains 2, 7, and 10 were recovered, individuals were hospitalized in high-complexity clinical care units, including intensive care, transplant, and pediatric hematology units.

**TABLE 1:** Summary of virulence traits of *P. frigoritolerans* strains

Trait	1 (ear swab)	2 (nail)	3 (urine)	4 (blood)	5 (central venous catheter)	6 (drainage)	7 (skin swab)	8 (urine)	9 (drainage)	10 (skin swab)
Genetic analysis										
Capsule	+									
Hemolysins										
Proteases										
Phospholipases										
Siderophores										
Holin-like toxin										
Zonula occludens toxin	−		+	−		+	−			
Phenotypic characterization										
Capsule	−									
Swimming	−			+	−					
Swarming	−			+	−					
Biofilm	−			+				−		
Hemolysins	−									
Proteases	−			+	−	+	−			
Phospholipases	−			+	−		+	−		
PCR amplification										
*bceT*	−									
*cytK*	−						+	−		
*entFM*	−									
*entS*										
*hblA*										
*hblC*										
*hblD*	−					+	−	+	−	
*nheA*	−			+	−		+		−	
*nheB*	−									
*nheC*										
*piplc*										
*sph*										

*Bacillus cereus* ATCC 14579 and ATCC 10987 and *Pseudomonas aeruginosa* ATCC 27853 were used as controls throughout the study. *Acinetobacter baumannii* ATCC 19606, *Aspergillus brasiliensis* ATCC 16404, *B. cereus* ATCC 14579, *Bacillus subtilis* ATCC 6051, *Candida albicans* ATCC 10231, *Enterococcus faecalis* ATCC 29212, *Escherichia coli* ATCC 25922, *Klebsiella pneumoniae* ATCC 700603, *Listeria monocytogenes* ATCC 13932, *P. aeruginosa* ATCC 27853, *Saccharomyces cerevisiae* ATCC 9469, *Salmonella enterica* serovar Typhimurium ATCC 14028, *Staphylococcus aureus* ATCC 12600, *Staphylococcus epidermidis* ATCC 35984, and *Trichosporon mucoides* ATCC 204094 were used to test the antimicrobial activity of the *Peribacillus* isolates. A clinical isolate of *Fusarium* spp. was also included.

### Colony morphology and microscopic characterization

Isolated colonies were spotted onto Columbia medium supplemented with 5% (vol/vol) sheep blood agar (COS; BioMérieux, Italy), brain heart infusion medium (BHI; Thermo Fisher Scientific, USA) supplemented with 1% (wt/vol) glucose (BHIG) agar, trypticase soy agar (TSA; PanReac AppliChem, Germany), and Luria-Bertani (LB; LLG Labware, Germany) agar plates. Plates were incubated for 48 h at 37°C, and colony morphology was observed at the end of the incubation time.

Bacterial cells were observed after Gram staining under an optical Olympus BX40 microscope (Olympus, Japan) at 1,000× magnification, and cell dimensions were measured. In parallel, India ink staining was performed to investigate capsule presence.

### MALDI-TOF MS and 16S rRNA gene sequencing

Matrix-assisted laser desorption/ionization-time of flight mass spectrometry (MALDI-TOF MS) was performed using a MALDI Biotyper Sirius mass spectrometer (Bruker, USA). Different colonies grown on LB agar plates were spotted on the MALDI plate and prepared as previously reported (19). A score ≥2.00 was considered valid for the correct identification at the species level.

Genomic DNA was extracted from each strain by the phenol-chloroform method as previously described (19). The universal primers 27F (5′-gagagtttgatcctggctcag-3′) and 1495R (5′-ctacggctaccttgttacga-3′) (20) were used for whole 16S rRNA gene amplification. Sanger sequencing was performed on purified 16S rRNA gene amplicons by Microsynth (Microsynth AG, Switzerland). Sequences were aligned by BLASTn (https://tinyurl.com/zftyc934), and the obtained consensus sequences were run against the SILVA (version 138.2) and BLAST (version 2.16.1+) databases for taxonomic resolution.

### Whole-genome sequencing and genome analysis

Whole-genome sequencing (WGS) was performed by PreBiomics (Italy) on the extracted genomes using Illumina NovaSeq 6000 DX (2 × 150 PE). The quality of sequenced samples was checked using TrimGalore (version 0.6.6; https://tinyurl.com/c9dadub3), and reads with a quality score <20, short reads (length <75 bp), and reads with more than five ambiguous nucleotides were removed. Assembly of the sequencing reads was performed using SPAdes (v4.0.0) (21). Species-level genome bin (SGB) identification of the assembled genomes was performed using PhyloPhlAn (v3.1) (22) against the Jun23 genomic database. All available reference genomes and metagenome-assembled genomes (MAGs) of *Peribacillus* spp. were collected from the MetaPhlAn 4 database for phylogenetic analysis (23). Phylogenetic reconstruction of the sequenced genomes, reference genomes, and MAGs was performed using Roary (24) and RaxML (25). Phylogenetic tree visualization was performed using the iTOL web service (v7; https://itol.embl.de/). Digital DNA-DNA hybridization (dDDH) was performed by uploading genomes on the Type (Strain) Genome Server (TYGS; https://tinyurl.com/2njvw9cy) of the Leibniz Institute DSMZ (26) to confirm previous SGB identification.

Assembled genomes of the clinical isolates and 21 complete genomes of *P. frigoritolerans* found on the NCBI genome database (https://tinyurl.com/yc8zb5bk) (i.e., strains DSM 8801, Ant232, BFRIG, C1141, CF20, CF29, CK6, GR2, HMB20428, IMGN5, JHS1, KF3, KF19, LC22, LN4, LSJ146, lyx31, NBTC-101, NS1, Q2H1, ZB201705) were annotated using Bakta (v1.8.2) (27). Plasmids and mobile elements were searched using PlasmidFinder (v2.0.1) (28) and mobileOG-db (v1.1.3) (29), respectively. Virulence factor profiling was performed using the online search tool of the VFDB database (30; https://www.mgc.ac.cn/VFs/search_VFs.htm). In addition, genes encoding virulence factors were manually searched in the annotated genomes using Proksee (https://proksee.ca/) by typing “capsule,” “hemolysin,” “protease,” “phospholipase,” “siderophore,” and “toxin.” Genes associated with antimicrobial resistance were investigated using the CARD Resistance Gene Identifier (v6.0.3) (31). Outputs DNA sequences of manually searched virulence factors and AMR genes from CARD were subsequently filtered for identity and coverage (>90%) using BLASTx (https://tinyurl.com/3sz6h6tp). BAGEL4 was used to detect genes encoding for bacteriocins and antimicrobial peptides (32), and resulting amino acid sequences were then aligned against the UniProtKB/Swiss-Prot protein database (https://www.uniprot.org/uniprotkb/).

### Growth in different conditions

Sixteen-hour bacterial cultures in LB broth were diluted (1:200) in 15 mL of fresh LB broth and incubated at 25°C or 37°C with constant shaking. Bacterial growth was monitored at 25°C or 37°C for up to 10 h by recording OD_600_. Psychrophily was tested on LB agar plates incubated for 7 days at 4°C, while thermotolerance on plates incubated for 48 h at 45°C. LB agar plates supplemented with 3%, 4.5%, 6%, or 7.5% (wt/vol) NaCl or adjusted at different pH values (i.e., 3, 5, 10, and 12) were used to test halotolerance and pH tolerance, respectively. Growth under anaerobic conditions was evaluated on LB agar plates incubated in anaerobic atmosphere (AnaeroGen Compact sachets; Thermo Fisher Scientific). The presence of colonies was evaluated after incubating for 48 h at 37°C.

### Biochemical characterization

Catalase production was evaluated by dropping hydrogen peroxide directly on colonies. The presence of cytochrome oxidase was assessed using oxidase test strips (Merck KGaA, Germany). *B. cereus* ATCC 14579 and *P. aeruginosa* ATCC 27853 were used as positive controls for catalase and oxidase tests, respectively.

Growth in 1.5% (wt/vol) agar or gelatin in the absence of other nutrients was evaluated by spotting colonies and incubating them at 37°C for 24 h. The ability to metabolize arabinose, fructose, glucose, lactose, mannitol, raffinose, sucrose, starch, mucin, casein, and inulin was tested in 96-well microplates. Ten microliters of bacterial suspensions (OD_600_ ~1) were added to 240 µL of each sterile compound (1.5% [wt/vol] in PBS). Microplates were incubated at 37°C for 24 h with constant shaking, and OD_600_ was measured using a Multiskan FC reader (Thermo Fisher Scientific).

### Sporulation assay

Schaeffer’s sporulation medium (30 mL) (33) was inoculated with 150 µL of 16-hour bacterial suspensions grown in LB broth. Cultures were incubated for 4 days at 30°C with constant shaking. After incubation, the suspensions were centrifuged at 4,000 × *g* for 10 min at 4°C and washed three times with cold spore washing buffer (34). Pellets were suspended in 5 mL of spore washing buffer, and two 1 mL aliquots were taken from each suspension. An aliquot was thermally treated at 80°C for 15 min to eliminate residual vegetative cells. Thermally treated and untreated aliquots were serially diluted 10-fold in PBS and seeded on LB agar plates. Plates were incubated at 37°C for 24 h, and the number of CFUs/mL was determined. *B. cereus* ATCC 10987 was used as a positive control. Sporulation percentage was calculated as: % = *n*° spores/*n*° vegetative cells × 100. For strains that did not sporulate within 4 days, sporulation experiments were prolonged up to 8 days. Three biological replicates, each with two technical replicates, were performed.

### Motility and biofilm formation

Bacterial motility was evaluated by light microscopy and by swimming and swarming plate assays, as previously described (35). The ability to form biofilms at 24 h and 48 h was determined in LB broth by the crystal violet assay (35). The OD_570_ values of the positive control and tested strains were adjusted by subtracting the values obtained from negative controls (sterile LB). Three biological replicates, each with two technical replicates, were performed. *B. cereus* ATCC 10987 was used as the positive control for all assays.

### Enzymatic activities, hemolysis, and virulence factors

Secretion of phospholipases, proteases, and hemolysins was evaluated by spotting colonies on agar plates supplemented with 2% (vol/vol) sterile egg yolk, 1.5% (wt/vol) skim milk, or 5% (vol/vol) sheep blood, respectively (35). The presence of halos around colonies was indicative of the presence of enzymatic or hemolytic activities. *B. cereus* ATCC 14579 was used as the positive control.

Since members of the genus *Peribacillus* are closely related to those of the genus *Bacillus*, the presence of *B. cereus* virulence factor-encoding genes (enterotoxin T, *bceT*; cytotoxin K, *cytK*; enterotoxin FM, *entFM*; enterotoxin S, *entS*; hemolysin BL [HBL], *hblA*, *hblC*, and *hblD*; non-hemolytic enterotoxin [NHE], *nheA*, *nheB*, and *nheC*; phosphatidylinositol-specific phospholipase C [PI-PLC], *piplc*; sphingomyelinase [SMase], *sph*) was assessed by PCR. PCR reactions were performed on genomic DNA extracted from *P. frigoritolerans* strains, as previously reported (35). PCR products were visualized after electrophoresis on a 1% (wt/vol) agarose gel. *B. cereus* ATCC 14579 was used as the positive control for each amplification.

### Antimicrobial susceptibility testing

Broth microdilution was performed according to EUCAST guidelines (36) using ITGP100 panels (for Gram-positive bacteria, Bruker) (37). Panels were incubated at 37°C for 24 h, and minimal inhibitory concentrations (MICs) were determined. MIC50 and MIC90 values, referring to the MICs that inhibit the growth of 50% and 90% of the tested microorganisms, respectively, were also calculated. Since no breakpoints are currently available for *Peribacillus* spp., susceptibility or resistance was defined using the updated CLSI and EUCAST breakpoints for *Bacillus* spp. (CLSI, 38, 39). When CLSI and EUCAST interpretation criteria differed, both interpretations were specified.

### Secretion of antimicrobial compounds

Antimicrobial activity was tested against a selection of bacteria and fungi using the agar overlay assay. *Peribacillus* colonies were picked and spotted on LB agar plates, and plates were incubated at 37°C for 16 h. The pre-incubated colonies were overlaid with 5 mL of LB or Sabouraud (Thermo Fisher Scientific) supplemented with 0.3% (wt/vol) granulated agar containing 10^8^ (for bacteria) or 10^6^ (for fungi) CFUs/mL of the selected strains (40). Plates were incubated at room temperature for 24 h, and the presence of inhibition halos upon the pre-incubated colonies, indicating antimicrobial activity, was evaluated.

## RESULTS

### Identification and genomics of the *P. frigoritolerans* strains

**Fig 1:**
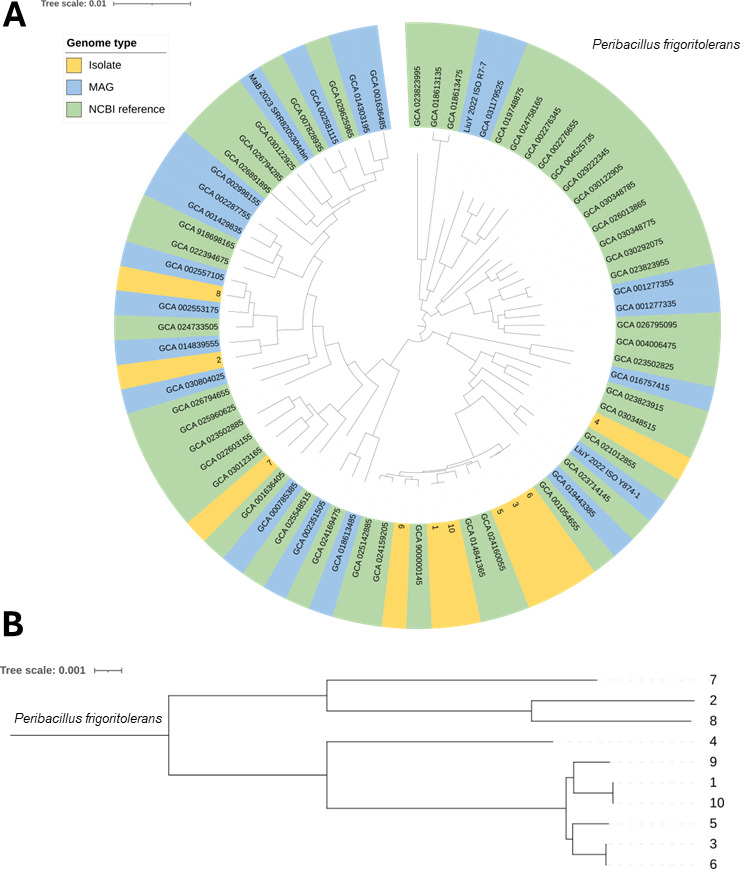
(**A**) Phylogenetic tree of *Peribacillus frigoritolerans*, including the strains isolated in this study (yellow), NCBI genome references (green), and MAGs (blue). (**B**) Phylogenetic tree of the 10 strains of *P. frigoritolerans* isolated in this study.

MALDI-TOF mass spectrometry identified all strains as *Peribacillus simplex*, along with the note: “Species *butanolivorans*/*frigoritolerans*/*muralis*/*simplex* from the genus *Peribacillus* display very similar profiles: for this reason, distinguishing these species is challenging.” Similarly, sequencing of the whole 16S rRNA gene did not identify the isolates at the species level due to the genetic similarity within the genus *Peribacillus*. In fact, analysis of the sequences against the SILVA database always resulted in *Peribacillus* spp., whereas alignments against the BLAST database matched with high identity (>99%) to several strains of *P. simplex* and *P. frigoritolerans* and, to a lesser extent, to *Peribacillus castrilensis* and *Peribacillus muralis*. All strains were definitely identified by WGS (i.e., SGB identification, ANI analysis, and dDDH) as *Peribacillus frigoritolerans* (sequencing coverage, 200–385×; ANI, ≥97.2%; average distance, ≤0.028; d_4_, ≥74%). Phylogenetic trees are reported in Fig. 1.

Genomes of the 10 *P. frigoritolerans* strains were as large as 5.4–5.9 Mbp. Their GC content was 40.2%–40.6%, and annotated coding sequences were 5,311–5,889. No plasmids were identified. On the contrary, several mobile genetic elements responsible for recombinations, integrations, and excisions, and transpositions were detected.

### Growth, metabolism, and sporulation

**Fig 2:**
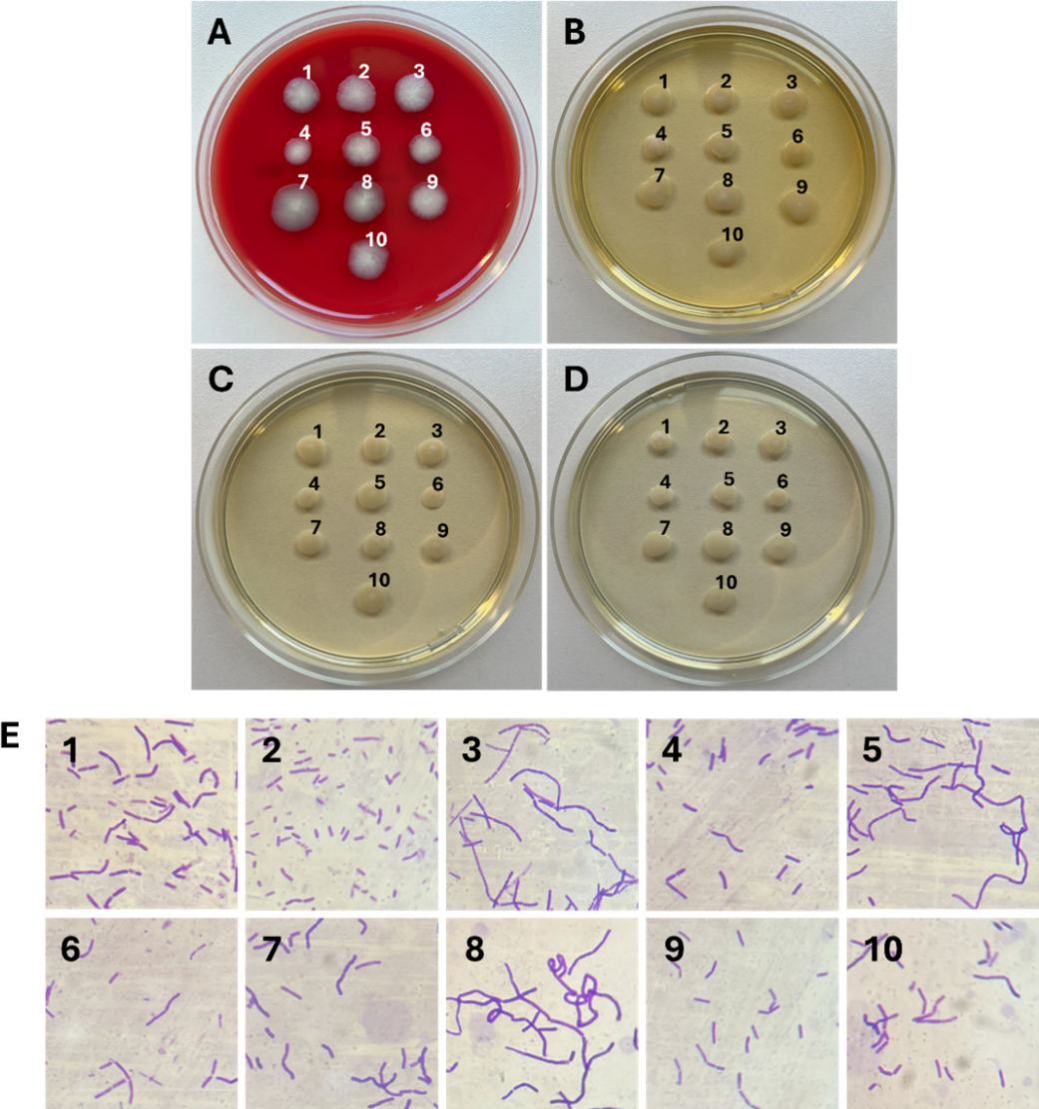
Colony morphology of *P. frigoritolerans* isolates grown on (**A**) COS, (**B**) BHIG, (**C**) TSA, and (**D**) LB agar. (**E**) Cell morphology and organization of *P. frigoritolerans* isolates stained by Gram staining (1,000× magnification).

A full biological characterization of the *P. frigoritolerans* isolates was performed. The *P. frigoritolerans* isolates were able to grow in all tested media, with the largest colonies being obtained on COS plates (Fig. 2A through D). While colonies appeared cream-colored on BHIG, TSA, and LB agar plates (Fig. 2B through D), they were white to gray on COS plates (Fig. 2A). Microscopically, they appeared rod-shaped and Gram-positive, with overall cell dimensions ranging from 2 to 5 µm (Fig. 2E). Strains 3, 5, and 8 were more prone to forming medium- to long-length chains than other strains, which were usually observed in single cells, pairs, or short chains (Fig. 2E). Although genes encoding capsule components were found in the genomes of all isolates (Table 1), none of the strains exhibited a capsule under the tested culture conditions.

**Fig 3:**
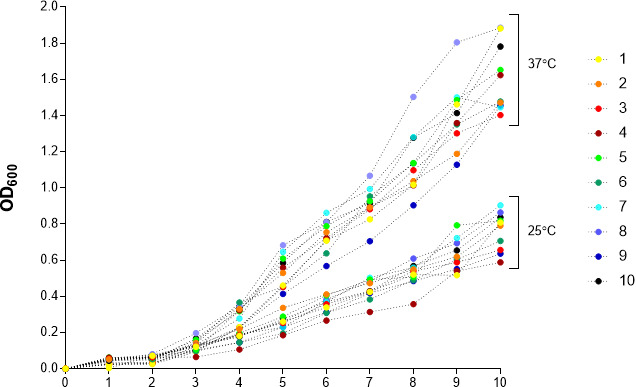
Growth of *P. frigoritolerans* strains at 25°C and 37°C in LB broth.

Monitoring growth at two different temperatures demonstrated that all strains replicated better at 37°C than at 25°C, with OD_600_ values ranging from 1.403 (strain 3) to 1.886 (strain 8) after 10 h at 37°C and from 0.588 (strain 4) to 0.904 (strain 7) at 25°C (Fig. 3). Strains 2, 4, 5, 7, 8, and 10 were able to grow at 4°C, although they formed only very modest colonies after 7 days of incubation. Strains 1, 2, 6–8, and 10 grew at 45°C. Therefore, strains 2, 7, 8, and 10 displayed a wide range of growth temperatures, ranging from 4°C to 45°C.

All isolates were able to grow in the presence of 3% and 4.5% NaCl, with the exception of strains 1 and 6 in the latter condition. In contrast, they did not grow in 6% and 7.5% NaCl. While all isolates grew at pH 5, none was able to grow at pH 12. Strains 2, 4, 5, 7, and 9 tolerated pH 3 well, and strains 1–5 and 7–9 survived at pH 10.

All strains resulted positive for catalase production and negative for cytochrome oxidase activity. In addition, they were able to grow under anaerobic conditions and, surprisingly, in the presence of agar or gelatin alone, as well as to metabolize all the tested carbon sources, thus confirming their versatile metabolism.

The ability to sporulate under *in vitro* favorable conditions was assessed. Strains 1, 4, 5, 7, and 10 sporulated at different percentages (30.1%, 87.5%, 19.4%, 57.7%, and 21.1% respectively) within 4 days. Interestingly, strain 8 displayed a sporulation rate of 100% at 4 days, similar to the positive control. On the contrary, we were unable to demonstrate sporulation for strains 2, 3, 6, and 9 under the tested conditions, even after prolonging incubation up to 8 days.

### Virulence potential

**Fig 4:**
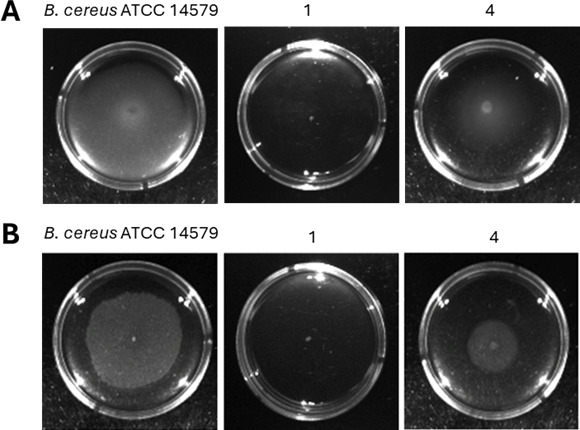
(**A**) Swimming halos in *Bacillus cereus* ATCC 14579 (positive control) and strain 4. (**B**) Swarming halos in *B. cereus* ATCC 14579 (positive control) and strain 4. Strain 1 was negative to both swimming and swarming motilities and was selected as a representative non-motile strain.

Proficiency in swimming and swarming, as well as in biofilm formation, is a crucial property for the colonization of different environments, including those found in the human host. Only strain 4 was able to swim and swarm in tested conditions (Table 1), with halo diameters of 20 mm and 18 mm, respectively (Fig. 4). Strain 4 also formed biofilms on polystyrene as early as 24 h (1.38 ± 0.02), which remained stable after 48 h of incubation (1.42 ± 0.05). Strains 5 (0.40 ± 0.02), 6 (0.68 ± 0.04), and 7 (0.35 ± 0.02) formed low-biomass biofilms only at 48 h (Table 1). The remaining strains did not form biofilms on polystyrene even after prolonged times.

As regards secreted virulence factors, genes encoding proteases, phospholipases, and siderophores were found in the genomes of all strains, as well as genes encoding a toxin (similar to the anthrax toxin lethal factor-related metallo-endopeptidase, identity ≥97.1%, coverage 100%) and a holin-like toxin (identity ≥96.7%, coverage 97%) (Table 1). In contrast, alignment with VFDB did not provide any output. Phenotypically, only strains 4 and 6 were able to actively secrete proteases, whereas only strains 4 and 7 were able to secrete phospholipases. None of the strains displayed hemolytic activity (Table 1).

Most of the genes encoding *B. cereus* virulence factors (i.e., *bceT*, *entFM*, *entS*, *hblA*, *hblC*, *nheB*, *nheC*, *piplc*, *sph*) were not detected in the *P. frigoritolerans* isolates. However, strain 7 was positive for *cytK*, potentially producing cytotoxin K; strains 6 and 8 were positive for *hblD*; and strains 4, 7, and 8 were positive for *nheA* (Table 1). Considering that HBL and NHE are trimeric toxins that, in *B. cereus*, are active only when the three subunits are present, it is reasonable to hypothesize that the same may occur in *Peribacillus* spp. and that these strains were not able to produce active HBL and NHE.

### Antibiotic resistance

The genomic analysis revealed that clinical isolates of *P. frigoritolerans* possessed genes associated with antibiotic resistance. In particular, *BcIII* (class A *B. cereus* β-lactamase), *FosBx1* (fosfomycin thiol transferase), *ereA* (erythromycin esterase), and *qacJ* (antibiotic efflux pump) were detected in all strains (identity ≥92.1%, coverage ≥99%). *vanR* and *vanY* in *vanF* cluster (glycopeptide resistance gene cluster, both found in 6 out of 10 isolates), *msrG* (mrs-type ABC-F protein, 5/10), and *dfrG* (trimethoprim resistant dihydrofolate reductase, 1/10) were variably present (identity ≥93.2%, coverage ≥92%).

A panel of antibiotics commonly used in healthcare settings for treating infections by Gram-positive bacteria was used to assess phenotypic antibiotic susceptibility. MIC values for each antibiotic are reported in Table 2. All *P. frigoritolerans* isolates displayed concordant results for a subset of antibiotics. They were all susceptible to gentamicin, linezolid, rifampicin, and vancomycin, as well as were resistant to ampicillin. Interpretations for levofloxacin differed based on CLSI/EUCAST breakpoints; all strains were classified as susceptible according to CLSI, but as susceptible only at increased exposure according to EUCAST. All strains were susceptible to clindamycin (or, in some cases, intermediate according to CLSI), with the exception of strain 4 (>1 µg/mL), while strains 3 (4 µg/mL), 6 (4 µg/mL), and 10 (2 µg/mL) were intermediate according to CLSI or resistant according to EUCAST to erythromycin. Overall, strains 3–6 displayed the highest MIC values and more critical resistance profiles.

**TABLE 2:** MIC, MIC50, and MIC90 values (µg/mL) of 25 antibiotics against the *P. frigoritolerans* strains^*c*^^,^^*d*^^,^^*e*^

Drug	1	2	3	4	5	6	7	8	9	10	MIC50	MIC90
β-Lactams												
AMP	0.5 **R^*a*^**		>8 **R^*a*^**	0.5 **R**^*a*^	4 **R^*a*^**	8 **R^*a*^**	0.5 **R^*a*^**				0.5 **R^*a*^**	8 **R^*a*^**
BPR	≤0.25		2	≤0.25	2	1	≤0.25		0.5	≤0.25	≤0.25	2
CPT	0.5		4	0.5	2		≤0.25	0.5			0.5	2
FOX	≤4										≤4	
OXA	≤0.25	1		≤0.25	1	≤0.25		0.5		≤0.25	≤0.25	1
SAM	≤2			4	≤2						≤2	
Glycopeptides												
DAL	0.25	≤0.06	0.12	≤0.06	0.25	≤0.06			0.25		≤0.06	0.25
TEC	≤0.25										≤0.25	
VAN	≤0.5 **S^*a*^**^,*b*^										≤0.5 **S^*a*^**^,*b*^	
Tetracyclines												
DOX	≤0.5										≤0.5	
TGC	≤0.12		0.25	0.5	≤0.12						≤0.12	0.25
Oxazolidinones												
LZD	≤1 **S^*b*^**										≤1 **S^*b*^**	
TZD	>1										>1	
Fluoroquinolones												
LVF	≤1 **S^*a*^**/**I**^*b*^										≤1 **S^*a*^**/**I^*b*^**	
MXF	≤0.25		1		≤0.25		1	≤0.25			0.5	≤0.25
Others												
CLI	1 **I^*a*^**/**S^*b*^**	0.25 **S^*a*^**^,*b*^	0.5 **S^*a*^**^,*b*^	>1 **id^*a*^**/**R^*b*^**	1 **I^*a*^**/**S^*b*^**	0.25 **S^*a*^**^,*b*^	≤0.12 **S^*a*^**^,*b*^	1 **I^*a*^**/**S^*b*^**			1 **I^*a*^**/**S^*b*^**	
DAP	≤0.25			0.5	≤0.25						≤0.25	
ERC	≤4/0.5										≤4/0.5	
ERY	≤1 **id^*a*^**^,*b*^		4 **I^*a*^**/**R^*b*^**	≤1 **id^*a*^**^,*b*^		4 **I^*a*^**/**R^*b*^**	≤1 **id^*a*^**^,*b*^			2 **I^*a*^**/**R^*b*^**	≤1 **id^*a*^**^,*b*^	4 **I**^*a*^/**R^*b*^**
FA	0.5	≤0.25	0.5	≤0.25	1		≤0.25	0.5			0.5	1
GEN	≤0.25 **S^*a*^**										≤0.25 **S^*a*^**	
MUP	≤1										≤1	
NIT	≤16										≤16	
RIF	≤0.06 **S^*a*^**	0.12 **S^*a*^**		≤0.06 **S^*a*^**	0.12 **S^*a*^**				≤0.06 **S^*a*^**	0.12 **S^*a*^**	0.12 **S^*a*^**	
**SXT**	≤0.25/4.75 **S^*a*^**										≤0.25/4.75 **S^*a*^**	

^*a*^ According to CLSI breakpoints.

^*b*^ According to EUCAST breakpoints.

^*c*^ S, susceptible; I, intermediate (CLSI)/susceptible at increased exposure (EUCAST); R, resistant; id, indeterminate.

^*d*^ AMP, ampicillin (0.25–8 µg/mL); BPR, ceftobiprole (0.25–2 µg/mL); CPT, ceftaroline (0.25–4 µg/mL); CLI, clindamycin (0.12–1 µg/mL); DAL, dalbavancin (0.06–0.5 µg/mL); DAP, daptomycin (0.25–2 µg/mL); DOX, doxycycline (0.5–4 µg/mL); ERC, erythromycin/clindamycin (4/0.5–8/1.5 µg/mL); ERY, erythromycin (1–4 µg/mL); FA, fusidic acid (0.25–1 µg/mL); FOX, cefoxitin (4–8 µg/mL); GEN, gentamicin (0.25–4 µg/mL); LVX, levofloxacin (1–4 µg/mL); LZD, linezolid (1–4 µg/mL); MUP, mupirocin (1–256 µg/mL); MXF, moxifloxacin (0.125–0.5 µg/mL); NIT, nitrofurantoin (16–64 µg/mL); OXA, oxacillin (0.25–2 µg/mL); RIF, rifampicin (0.06–1 µg/mL); SAM, ampicillin/sulbactam (2–8 µg/mL); SXT, trimethoprim/sulfamethoxazole (0.25/4.75–4/76 µg/mL); TGC, tigecycline (0.12–0.5 µg/mL); TZD, tedizolid (0.5–1 µg/mL); TEC, teicoplanin (0.25–4 µg/mL); VAN, vancomycin (0.5–4 µg/mL).

^*e*^ S/I/R were in bold to highlight the presence of interpretation by CLSI or EUCAST.

### Antimicrobial activity

Since some of the strains were isolated from body sites that naturally harbor diverse microbial communities (i.e., skin, genitourinary tract), we assessed whether they produced antimicrobial compounds that could modify existing microbiota and facilitate colonization of a new environment.

Different genes encoding putative bacteriocins and antimicrobial peptides were identified. In particular, strains 1, 3, 5, 6, 9, and 10 possessed the gene encoding a sactipeptide (57–60 aa) partially matching with the sporulation killing factor SkfA from *Paenibacillus larvae* subsp. *larvae* DSM 25430 (61 aa, identity 60–63.2%). Strain 4 displayed the gene encoding a bacteriocin (73 aa) similar to an uncharacterized bacteriocin from *Bacillus thuringiensis* ATCC 10792 and bacteriocin UviB from *Bacillus thuringiensis* subsp. *israelensis* ATCC 35646 (76 aa, identity 57.1%). The gene encoding a lasso peptide (40–43 aa), similar to paeninodin from *Pullulanibacillus camelliae* CGMCC 1.15371 (43 aa, identity 50%), was found in strains 5 and 9. No genes associated with bacteriocin/antimicrobial peptide production were detected in remaining strains.

*Peribacillus* isolates were tested against Gram-positive and Gram-negative bacteria, as well as yeasts and molds, using agar overlay assays. All the strains were active against *B. cereus*, but strains 1, 4, 7, 8, and 10 were further active against *B. subtilis*, *E. faecalis*, *L. monocytogenes*, *S. aureus*, and *S. epidermidis*. Interestingly, the same strains displayed antimicrobial activity also against *C. albicans*, *A. brasiliensis*, and *Fusarium*. In contrast, none of the strains exhibited activity against Gram-negative bacteria, *S. cerevisiae*, or *T. mucoides*.

### Functional pangenome analysis

A functional pangenome analysis was carried out including our 10 clinical isolates and 21 environmental *P. frigoritolerans* strains whose genomes were available in the NCBI database. The genomic analysis demonstrated that most of the annotated genes related to antibiotic resistance were prevalent in the *P. frigoritolerans* population, including *BcIII* (30/31), *FosBx1* (31/31), *ereA* (31/31), *vanR* and *vanY* in *vanF* cluster (21/31 and 23/31, respectively), *qacJ* (31/31), and *msrG* (13/31). Interestingly, gene dimensions were sometimes different across the strains (i.e., 933, 945, 948, or 951 bp long *BcIII*). Conversely, the frequency of other genes, such as *dfrG* (4/31), *qacG* (3/31), *ereB* (type II erythromycin esterase, 2/31), *clcD* (23S subunit methyltransferase, 1/31), and *mphM* (macrolide phosphotransferase, 1/31), was rather rare.

As for the clinical isolates, the same genes encoding secreted virulence factors (i.e., proteases, phospholipases, siderophores, toxins) were detected in all the environmental strains.

Extending the genomic analysis to environmental strains revealed no additional bacteriocin- and antimicrobial peptide-encoding genes beyond those already identified in clinical isolates, but only slight differences in gene length. The gene encoding the sactipeptide was found in nine out of 31 strains (i.e., 1, 3, 5, 6, 9, 10, Ant232, BFRIG, C1141), while that encoding the uncharacterized bacteriocin was present in four out of 31 strains (i.e., 4, KF19, LSJ146, Q2H1). The lasso peptide was potentially encoded by six out of 31 strains (i.e., 5, 9, IMGN5, JHS1, LSJ146, NBTC-101). Overall, 15 out of 31 genomes did not display genes associated with bacteriocin synthesis.

## DISCUSSION

Most of the knowledge on *P. frigoritolerans* relates to its biotechnological and agricultural applicability, but understanding the physiology and pathogenic potential of this species is still very limited. This lack becomes particularly important when *P. frigoritolerans* is isolated from clinical specimens. Is the isolation of such bacterium clinically relevant, or should it be simply considered as a contaminant or non-pathogenic bacterium? Could it effectively act as a pathogen in humans? In the present study, we sought to address these questions by assessing the physiology of ten *P. frigoritolerans* strains isolated from humans for the first time and evaluating key traits associated with pathogenicity.

The identification of *Peribacillus* spp. is generally complicated due to the absence of comprehensive biological and biochemical characterization of such species and the high similarities in 16S rRNA gene sequences and protein content. We provided new details on the physiology of this species, but, albeit relevant and novel for (almost) unknown microorganisms as in the case of *P. frigoritolerans*, cannot be helpful for identification purposes. Even molecular identification is challenging. In fact, both amplicon sequencing and MALDI-TOF MS may fail to properly identify bacterial isolates at the species level (4, 41), thus making taxonomic resolution difficult. Also in our case, MALDI-TOF MS and 16S rRNA gene sequencing were insufficient for identification. For this reason, we performed WGS, which definitely classified our strains as *P. frigoritolerans*. Therefore, WGS appears the most accurate method to identify *Peribacillus* spp., albeit rarely feasible in clinical practice, likely leading to an underestimation of *P. frigoritolerans* infection cases.

*P. frigoritolerans* displayed a highly flexible physiology and metabolism, with strains markedly differing in their capabilities and versatility, indicating that certain traits are strain-specific. Interestingly, all isolates grew faster at 37°C than at 25°C, indicating their preference for ecological niches more similar to those in humans and warm-blooded animals. Furthermore, most of the strains were psychrophilic, thermotolerant, and able to grow in a wide range of pH and NaCl concentrations. These findings highlight the extreme adaptability of this species to grow and persist in different environmental conditions, as already reported in previous studies (3, 42, 43). The metabolic variability among *P. frigoritolerans* isolates underlines again the importance of molecular characterization for identification purposes.

Spores of *Peribacillus* spp. are poorly investigated, unlike those from other clinically relevant *Bacillus*, *Clostridium*, and *Clostridioides* species (30, 44–46), and important details on sporulation, germination, and spore stress resistance are missing. The only information found in the literature concerns spores of the environmental *P. frigoritolerans* UJA_MA_369, which exhibit a peculiar exosporium with a potential role in bacterial virulence and interaction with the environment (41). Since the ability to form spores is crucial for bacterial survival, dissemination in the environment, and contamination of surfaces and objects, this piece of knowledge is relevant for preventing infections in clinical settings, especially in critical-care units (47). Here, we provided the first evidence that *P. frigoritolerans* strains exhibit different sporulation abilities. Most of the investigated strains formed spores under favorable *in vitro* conditions, while we were unable to demonstrate sporulation in a minority of them, even after prolonged incubation. However, this result is in line with previous studies reporting that not all strains belonging to genera *Bacillus* and *Clostridium* form spores under laboratory conditions (48, 49).

An extreme diversity also emerged when virulence factors were evaluated. Motility appears to be a rare property of our *P. frigoritolerans* isolates, since only one out of 10 strains resulted positive in swimming and swarming assays. Only some of the strains formed biofilms, produced proteases and phospholipases, and carried *B. cereus* toxin- and exoenzyme-encoding genes. These findings are in line with previous studies evidencing that motility, as well as biofilm, enzyme, and toxin production, is a strictly strain-specific property (15, 41, 43, 50). *Peribacillus* spp. have been previously shown to possess the *bceT*, *cytK*, *hblC*, *hblD*, *nheA*, and *nheC* genes (15, 51), but the encoded toxins were never detected in culture supernatants. Herein, we demonstrated that three *P. frigoritolerans* isolates possess *cytK*, *hblD*, or *nheA*, with only one potentially expressing cytotoxin K. Although genomic analysis evidenced the presence of genes related to capsule production and proteolytic and phospholipase activities, all our isolates did not exhibit capsule in the tested culture conditions, while only strains 4, 6, and 7 displayed proteolytic or phospholipase activities. The inconsistency between the genetic and phenotypic results may be due to incorrect gene expression or the absence of one or more genes required for the production of functional proteins or structures, but further studies are required to address this discrepancy.

Similarly, the presence of resistance genes did not often correlate with resistance phenotypes to specific antibiotics in our isolates. In fact, the antibiotic susceptibility testing showed that the isolated strains were generally sensitive to commonly prescribed antibiotics, including cephalosporins, glycopeptides, and fluoroquinolones, while being resistant to ampicillin and, occasionally, erythromycin and clindamycin. The phenotypic resistance of all strains to ampicillin, but interestingly not to other β-lactams, could be reasonably explained by the presence of the *BcIII* gene, which encodes a class A β-lactamase mainly active on penicillins. On the contrary, while all strains displayed the *ereA* gene encoding an erythromycin esterase, only a few were phenotypically resistant to erythromycin. Similarly, although the presence of genes associated with vancomycin resistance was present in all strains, phenotypic susceptibility to vancomycin was observed. In the case of *ereA* and *van* genes, the lack of correlation between genotypic and phenotypic outcomes is intriguing but presumably explainable by the absence of the full set of genes required for resistance or by altered gene expression. Antibiotic resistance of *Peribacillus* spp. was scarcely investigated in the literature. *P. frigoritolerans* WR1 isolated from lake water was found to possess *blaTEM*, *ampC*, and *qnrS* genes by PCR and to be phenotypically resistant to cefotaxime (52). Part of the genes constituting the *vanF* cluster were detected in *P. frigoritolerans* GD44 isolated from soil (53). Clinical isolates of *P. simplex* were susceptible to carbapenems, fluoroquinolones, linezolid, and vancomycin (17, 18), but were resistant to macrolides and lincosamides. The results from these studies are similar to those obtained for our *P. frigoritolerans* isolates, overall indicating that this species has not yet acquired alarming resistance phenotypes.

Some *P. frigoritolerans* strains demonstrated antimicrobial activities against common phytopathogens, including *Xylella fastidiosa*, filamentous fungi, and nematodes, through the production of antimicrobial compounds and the induction of systemic plant resistance (14, 50, 54, 55). A previous study also demonstrated that two *Peribacillus* strains were active against *B. cereus*, but not against *S. aureus*, *E. faecalis*, *Listeria innocua*, or Gram-negative bacteria, thus demonstrating a very narrow spectrum of action in that case (55). Besides the importance of *Peribacillus* as a biocontrol agent in agriculture, this interspecies competition may play a role in the colonization of new environments inhabited by other species or complex communities, such as human body sites where a resident microbiota is normally present. In fact, the introduction of a new species with a strong antimicrobial activity could determine a compositional shift in the local microbial populations, the overgrowth of certain species, and the possible onset of infections (56). Some of our strains, particularly those isolated from skin, urine, and blood, displayed antimicrobial activity against some Gram-positive bacteria and fungi, but not against Gram-negative bacteria. We hypothesize that the antimicrobial activity exhibited by these strains may have contributed to the establishment of infection through the alteration of the local microbiota. Again, the antimicrobial potential of *P. frigoritolerans* appears strictly strain-dependent. The presence of bacteriocin- and antimicrobial peptide-encoding genes in the genomes of the *P. frigoritolerans* strains isolated in this study corroborates their antimicrobial activity but does not exclude the involvement of other molecules.

In the end, an association between the overall virulence potential and infection site and severity can be hypothesized. In fact, strain 4, which exhibited the most aggressive virulence profile, had been able to penetrate in the bloodstream determining bacteremia. Similarly, strain 6, producing biofilms and proteases, was recovered from a drainage. Interestingly, in this study, biofilm production was frequently linked with colonization of long-term devices and infections in deep sterile sites, supporting the role of this virulence trait in more severe infections.

This is the first report of *P. frigoritolerans* isolation from human clinical samples. Altogether, the obtained data provide insights into the pathogenic potential of *P. frigoritolerans*, despite evident strain-specific differences in pathogenicity and virulence. Considering that systems used in the diagnostic routine cannot properly distinguish within the genus *Peribacillus* and the high inter-strain variability, *P. frigoritolerans* and *Peribacillus* spp. could be preventively considered as potential pathogens rather than just as environmental contaminants, depending on the clinical situation.

## Data Availability

Raw sequences from the *P. frigoritolerans* strains isolated in this study are publicly available at the European Nucleotide Archive (accession PRJEB104082). Other data sets generated herein will be made available from the corresponding author on reasonable request.
